# Social Media News Use Induces COVID-19 Vaccine Hesitancy Through Skepticism Regarding Its Efficacy: A Longitudinal Study From the United States

**DOI:** 10.3389/fpsyg.2022.900386

**Published:** 2022-06-10

**Authors:** Saifuddin Ahmed, Muhammad Ehab Rasul, Jaeho Cho

**Affiliations:** ^1^Wee Kim Wee School of Communication and Information, Nanyang Technological University, Singapore, Singapore; ^2^Department of Communication, University of California, Davis, Davis, CA, United States

**Keywords:** COVID-19, vaccine, social media, skepticism, United States

## Abstract

There are mounting concerns about the adverse effects of social media on the public understanding of the COVID-19 pandemic and its potential effects on vaccination coverage. Yet early studies have focused on generic social media use and been based on cross-sectional data limiting any causal inferences. This study is among the first to provide causal support for the speculation that social media news use leads to vaccine hesitancy among US citizens. This two-wave survey study was conducted in the US using Qualtrics online panel-based recruitment. We employ mediation and moderated mediation analyses to test our assumptions. The results suggest that using social media to consume news content can translate into vaccine hesitancy by increasing citizens’ skepticism regarding the efficacy of vaccines. However, these effects are contingent upon the news literacy of users, as the effects on vaccine hesitancy are more substantial among those with lower news literacy. The current study recommends to public policymakers and vaccine communication strategists that any attempt to reduce vaccine hesitancy in society should factor in the adverse effects of social media news use that can increase vaccine safety concerns.

## Introduction

During the COVID-19 pandemic, social media offered individuals affordable and easy access to large amounts of news and information. For instance, the hashtag #COVID19 was the top hashtag on Twitter, and was tweeted 400 million times in 2020, with #StayHome being the third biggest hashtag (McGraw, 2020). Individuals have increasingly relied on social media for information related to COVID-19 due to the mass availability of news on multiple platforms, a term coined as an “infodemic” by scholars (Cinelli et al., 2020).

During the pandemic, the world was increasingly physically distant, with social distancing measures, public gatherings disbanded, and remote work environments instituted in many countries worldwide. As a result, social media rose to fulfill a critical role as a source of social news and the primary information outlet of governments and health organizations. However, the news and informational use of social media related to COVID-19 occur in masse with its communicative use for buffering COVID-19-related stress and anxiety (Zhao and Zhou, 2021), sharing stories of hardship (Cauberghe et al., 2021), and its recreational use for sharing humorous content and memes related to the pandemic (Akram et al., 2021). Consequently, the infodemic has also raised concerns about the large-scale spread of low-quality and unverified information and even misinformation related to COVID-19 medical research.

The consequences of the infodemic are potentially severe. For instance, recent studies have suggested that the harmful effects of misinformation circulated on social media related to COVID-19 include dwindling trust in public health authorities and the effectiveness of COVID-19 prevention protocols (Lovari, 2020). In addition, previous scholars have attributed the distrust to the unabated spread of misinformation on social media platforms, with some individuals being more vulnerable to misinformation than others (Guess et al., 2020).

The harmful effects of social media use also extend to users’ behavior toward preventive medicine. For example, scholars have identified vaccine hesitancy as a significant challenge in the fight against COVID-19 (Courtney and Bliuc, 2021; Wang and Zhang, 2021). Unfortunately, recent research from Italy (Reno et al., 2021) and the United Kingdom (Chadwick et al., 2021) suggests increased social media use is associated with higher vaccine hesitancy. In addition, with the emergence of more transmissible COVID-19 variants, such as the Delta and Omicron variants, health misinformation may lead individuals to generate and develop false beliefs about the effectiveness of vaccines. This is somewhat evidenced in the fact that, despite the mass availability of vaccines in the US, only 63.7% of the population is fully vaccinated against COVID-19 (CDC Covid Data Tracker, 2022).

While the literature on social media use and COVID-19 vaccine hesitancy continues to grow, it suffers from three significant drawbacks. First, most scholars pay attention to general social media use, not news consumption behavior. It is critical to examine news consumption behavior precisely because reliance on social media platforms for news would make individuals more vulnerable to false news and information (Melki et al., 2021). Secondly, limited attention has been paid to identifying the mechanism through which social media use translates into vaccine hesitancy. Thirdly, the most recent findings are based on cross-sectional data that do not offer any causal inferences. It is difficult to conclude if social media use causes or is simply related to vaccine hesitancy.

**FIGURE 1:**
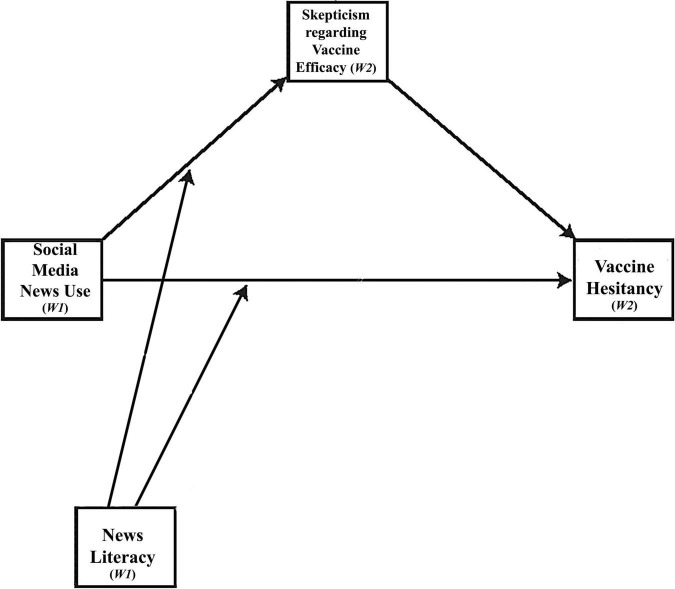
Conceptual framework.

This study offers a conceptual model that explains how social media news use causes vaccine hesitancy. Specifically, we argue that social media news use leads to vaccine hesitancy by inducing skepticism toward the efficacy of the vaccines (a mediated relationship). Further, we point out that this mediated mechanism can be moderated by news literacy such that the adverse effects of social media news use would be most substantial for those with lower levels of news literacy (Figure 1 illustrates the conceptual framework). Researchers have pointed out that news literacy is a proactive solution to combatting misinformation rather than a reactive solution, which has been the highlight of existing studies focusing on fighting misinformation (Vraga and Tully, 2021; Hameleers, 2022). To measure news literacy, we assess individual knowledge about media systems, industries, and their effects (see Vraga and Tully, 2021). News literacy requires the consumer to be mindful of the complex relationships between the origin of the news, its author, and the environment. Recent scholarship assessing the merit of news literacy as an intervention to correct misinformation has yielded positive results (Roozenbeek et al., 2020).

Therefore, to fill in the existing research gaps, this study relies on two-wave survey data (from the United States), allowing for causal inferences about the relationships between social media news use, skepticism about vaccines’ efficacy, and vaccine hesitancy. Also, it allows for an analysis of how news literacy moderates the discussed relationships. Ultimately, this study contributes meaningfully to a growing body of literature on understanding the effects of social media on vaccine hesitancy during the COVID-19 pandemic.

## Materials and Methods

### Participants

We employed the survey research firm Qualtrics LLC (Provo, UT, United States) to gather the data from their online panel. To increase the representativeness of the findings, we matched the sampling frame to US population metrics focusing on age and gender quotas (with respondents being adults between the age of 18 and 99 years). This approach has been frequently used to make conclusions about the broader population (Chadwick et al., 2021; Reno et al., 2021; Vraga and Tully, 2021). The first wave of the survey was conducted in late September 2020 (4 weeks before the US Presidential election), and the second wave was conducted right before the elections (30 October 2020 to 2 November 2020). Out of the 1,948 respondents in the first wave (W1), 673 respondents answered the survey during the second wave (W2: retention rate = 35%). The demographic details are included under the covariates section.

### Measurements

*COVID-19 vaccine hesitancy* was measured by asking respondents if a vaccine to prevent COVID-19 was available today, would they take it. The responses ranged from 1 = definitely get the vaccine to 4 = definitely not get it (W1: *mean* = 2.31; *SD* = 1.10; W2: *mean* = 2.22; *SD* = 1.05).

*Skepticism toward COVID-19 vaccine efficacy* was measured by asking respondents how much confidence they have that the research and development process will produce a vaccine for COVID-19 in the US that is *safe and effective*? The responses ranged from 1 = great deal of confidence to 4 = no confidence at all (W1: *mean* = 2.08; *SD* = 0.91; W2: *mean* = 1.96; *SD* = 0.87).

Social media news use was measured through the previously validated measure (Ahmed et al., 2021). Five items asked respondents how often (1 = never to 5 = daily) do they use the following features of social media to get news and information about social, political, and public affairs (sample items: post in their timeline about social, political, or public affairs news and read your news feed about social, political, or public affairs news). The responses for the five items were averaged to create a scale of social media news use (W1: *mean* = 2.21; *SD* = 1.21, α = 0.91).

*News literacy* was adapted from existing measures (Vraga and Tully, 2021) and measured through 9-item multiple-choice questions that tested participants’ knowledge of media structures and their effects. Sample item included a) people most often seek out news and information that: (1 = aligns with their views, 2 = differs from their views, 3 = requires effort to find out, and 4 = none of the above). The correct responses were coded as one and incorrect answers as zero. Following previously adopted approach (Vraga and Tully, 2021), we calculated news literacy by summing the score for correct responses (W1: *mean* = 4.66; *SD* = 2.29, KR-20 = 0.65; minimum = 0 and maximum = 9).

### Covariates

This study includes several covariates that may influence attitudes toward vaccines. These include demographics and motivational controls. Demographics include (a) age (*mean* = 45.79, *SD* = 16.66), (b) gender (52% females), (c) education (*Median* = Bachelor’s degree), (d) income (*Median* = $7,000–$8,999) and e) race (77% white). Motivational controls include news trust, political interest, political trust, political efficacy, partisanship, and traditional media news use. Details are included in Supplementary Material.

### Analysis

We employ autoregressive models to test the effect of social media news use on vaccine hesitancy. Over time, the change in patterns is estimated by regressing the second wave variable (vaccine hesitancy at wave two) on its corresponding wave one value (vaccine hesitancy at wave one). The demographics and motivational controls at wave one are also used as covariates. The autoregressive approach reduces error variance compared to the alternative method of changes in intra-attitudinal differences. We use PROCESS macro for SPSS (Hayes, 2018) to test the mediation and moderated mediation effects.

## Results

### Preliminary Cross-Sectional Observations

Before examining the autoregressive models, we explored the cross-sectional relationships between variables of interest and skepticism regarding vaccine efficacy and hesitancy to get the vaccine (see Table 1).

**TABLE 1:** Predicting skepticism regarding vaccine efficacy and vaccine hesitancy (cross-sectional observations).^a^

Cross-sectional observations	Vaccine efficacy skepticism (W1)	Vaccine hesitancy (W1)

	**β**	**β**
**Controls**		
Age	−0.122***	–0.022
Gender (1 = females)	0.118***	0.138***
Education	−0.119***	−0.140***
Income	0.001	–0.008
Race (0 = white)	−0.059**	−0.112***
Political interest	–0.015	–0.035
Political trust	−0.203***	−0.197***
Political efficacy	–0.022	0.042
Partisanship	–0.033	0.077***
Traditional media news use	−0.057*	−0.074**
News trust	−0.183***	−0.132***
**Independent variables**		
Social media news use	0.054*	–0.020
News literacy	−0.055*	0.033
**Total R** ^2^	0.227	0.216

^a^Statistical significance is marked as *p < 0.05; **p < 0.01; ***p < 0.001.

The results suggest that those with higher levels of political trust, traditional media news use, and news trust were not only less likely to be skeptical regarding the efficacy of the vaccines but also less likely to show hesitancy (political trust: skepticism, β = –0.203, *P* < 0.001; hesitancy, β = –0.197, *p* < 0.001; traditional media news use: skepticism, β = –0.057, *p* < *0.05*; hesitancy, β = –0.074, *p* < 0.01; news trust: skepticism, β = –0.183, *p* < 0.001; hesitancy, β = –0.132, *p* < 0.001).

In addition, social media news use (β = 0.054, *p* < 0.05) was positively associated with skepticism regarding vaccine efficacy and those with higher levels of news literacy were less skeptical (β = –0.055, *p* < 0.03). We also observe that the younger respondents (age, β = –0.122, *p* < 0.001) were more skeptical of vaccine efficacy, and those who leaned toward the Republican party (partisanship, β = 0.077, *p* < 0.001) were more hesitant to get the vaccine.

### Social Media News Use, Vaccine Skepticism, and Hesitancy: Longitudinal Observations

To examine the mechanism of how social media news use translates into COVID-19 vaccine hesitancy *via* inducing skepticism regarding the efficacy of the vaccine (indirect mediation effect), we ran a mediation model (Model 4) with a bootstrapping method (PROCESS: Hayes, 2018).

**FIGURE 2:**
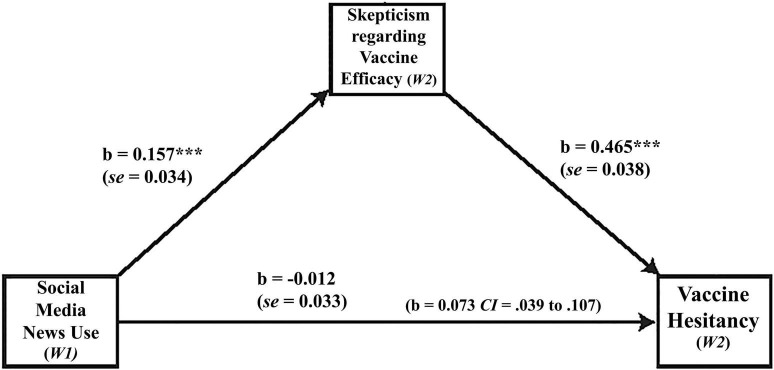
Indirect effects of social media news use (w1) on vaccine hesitancy through skepticism. ***p < 0.001, **p < 0.01; Estimates are calculated using the PROCESS macro for SPSS (Model 4; Hayes, 2018). The number in the parenthesis is the indirect effect with LLCI to ULCI. Bootstrap resample = 5,000. Statistical controls include *age*, *gender*, *education*, *income*, *race*, *political trust*, *political interest*, *political efficacy*, *partisanship*, *traditional media news use*, *news trust*, *and news literacy*.

The relationship between variables is illustrated in Figure 2. The results suggest that social media news use was positively associated with skepticism regarding the efficacy of vaccines (*b* = 0.157, *SE* = 0.034, *p* < 0.001) which in turn was positively associated with vaccine hesitancy (*b* = 0.465, *SE* = 0.038, *p* < 0.001). However, the direct relationship between social media news use and vaccine hesitancy was statistically insignificant (*b* = –0.012, *SE* = 0.033, *p* = 0.72).

Formal statistical testing for the mediation process, based on bootstrapping method, confirmed that the effect of social media news use on vaccine hesitancy is mediated by skepticism toward the vaccine. The indirect effect of social media news use on vaccine hesitancy through skepticism was statistically significant (*b* = 0.073, *SE* = 0.017, bootstrapping CI = 0.039–0.107). The direct effect of social media news use on vaccine hesitancy was statistically insignificant (*b* = –0.012, *SE* = 0.033, bootstrapping CI = –0.075–0.056). Thus, based on these results, we can infer that social media news use causes vaccine hesitancy but only through inducing skepticism regarding the efficacy of the vaccines.

### The Role of News Literacy: Longitudinal Observations

Next, we test how news literacy moderates the mediation process discussed above. We employed a conditional process analysis using the PROCESS macro (Model 8: Hayes, 2018). The index of moderated mediation was statistically significant (index = –0.014, *SE* = 0.007, LLCI = –0.029, ULCI = –0.002), suggesting that the mediation of social media news use through skepticism regarding the efficacy of vaccine on hesitancy varies depending on the levels of news literacy.

The patterns of indirect effect sizes indicate that the effect decreases with an increase in news literacy (News literacy at –1 SD: *b* = 0.102, *SE* = 0.023, CI = 0.058–0.148; at mean: *b* = 0.070 *SE* = 0.018, CI = 0.06–0.105, and at +1 SD: *b* = 0.038, *SE* = 0.024, CI = –0.012 to 0.084). Here, the strength of indirect effects *via* skepticism regarding vaccine efficacy decreases with an increase in levels of news literacy. Thereby, suggesting that the mechanism is most significant for those with lower levels of news literacy. See Supplementary Material for more details.

**FIGURE 3:**
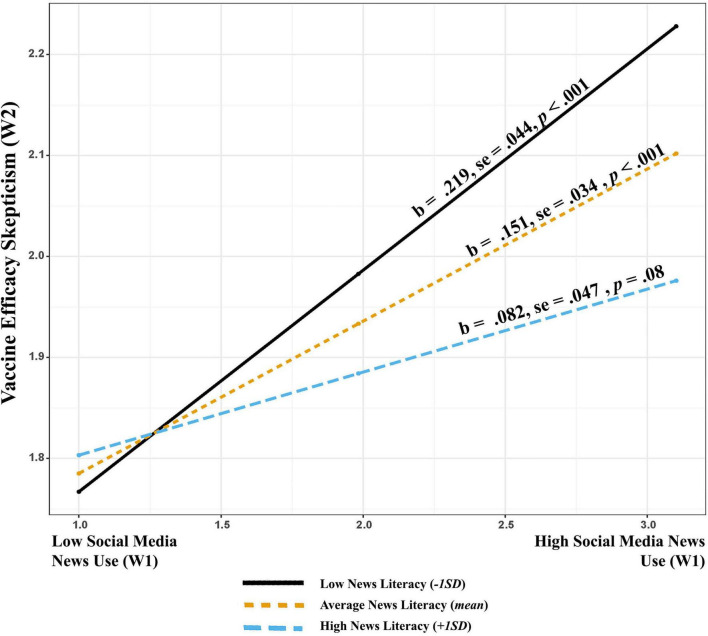
Conditional effect of social media news use (w1) at different values of news literacy (w1) predicting skepticism (w2).

Next, the interaction between social media news use and news literacy predicting skepticism regarding efficacy predicting was statistically significant (*b* = –0.031, *SE* = 0.014, *p* < 0.01). Probing the interaction through J-N technique suggests that the effects are significant for news literacy levels below 7.25 (82.27% of the sample). The interaction plotted in Figure 3 suggests that increased social media use induces greater skepticism regarding vaccine efficacy for individuals with lower levels of news literacy than those with moderate and higher levels of news literacy.

## Discussion

Most studies investigating the relationship between social media use and vaccine hesitancy have relied on cross-sectional data and do not offer a mechanism for how use translates into vaccine hesitancy (Chadwick et al., 2021; Reno et al., 2021; Ruiz and Bell, 2021). This study is a rare effort and is one of the first to provide causal support for the speculation that social media news use leads to vaccine hesitancy among US citizens. Overall, the findings suggest that increased news use *via* social media platforms induces skepticism regarding vaccines’ efficacy, consequently translating into vaccine hesitancy. Furthermore, we find that these effects are more substantial among those with lower than higher levels of news literacy. Therefore, the study highlights that the adverse impact of social media is contingent upon news literacy.

The findings of this study are particularly salient in the context of the COVID-19 pandemic within the US. The nation claimed the greatest number of vaccines globally and was one of the first countries to administer population-wide vaccine deployment. Yet, 2 years after the outbreak, it has also witnessed several anti-vaccine campaigns, and a significant portion of the population (39 million) remains unvaccinated (Healy et al., 2021). More recently, the surge in cases driven by the Omicron variant in the US is being dubbed as the “pandemic of the unvaccinated” (Barone, 2022). While government and media outreach has continuously engaged in vaccine communication, recent reports suggest millions choose to remain unvaccinated despite the infectiousness of some variants. We recommend several approaches to manage vaccine communication and hesitancy induced by social media.

First, our findings show that news literacy can have a buffering effect against vaccine hesitancy. The results are not surprising, given that higher news literacy levels would allow social media news users to distinguish between real and false news and accordingly judge the safety of COVID-19 vaccines. However, given that the US largely scores low on news literacy (Carr, 2021), it is disquieting that a significant proportion of those who rely on social media for news consumption with moderate and lower levels of news literacy will be prone to questioning the efficacy of vaccines. While the government and many civil society organizations continue to employ programs to inform the public regarding the efficacy details of the vaccines, the findings also suggest the need to build news literacy, especially among those who rely on social media platforms as their primary source of news.

Second, the role of partisanship is widely debated in vaccine hesitancy. While our cross-sectional analyses support existing literature showing that those who identify as Republicans show more significant vaccine hesitancy than others, additional moderated mediation analyses did not find any significant effects of partisanship on the mechanism discussed here (results not included for brevity). Simply put, the mechanism discussed in this study (social media news use > skepticism regarding vaccine efficacy > vaccine hesitancy) is independent of partisanship effects.

To interpret the study’s findings, it is necessary to consider that the data was collected during an evolving event and an ongoing global pandemic.

Firstly, because this study utilizes survey data from the early days of the pandemic, the findings help interpret what the early effects of news use may have been like. Since then, the COVID-19 information environment has progressed. Therefore, the results will add to any study conducted in the future. In addition, agent-based models, experiments, or year-on-year surveys that theorize or account for peer effects, economic and policy changes, and other exogenous shocks may also explain how vaccine hesitancy trends could evolve.

Secondly, the findings are based on the US. However, we cannot generalize the findings to other societies without definite empirical examinations. It will be noteworthy to explore if the results apply to different democratic contexts with similar levels of vaccine deployment and social media penetration.

Thirdly, we use a quota-based online panel survey which is increasingly accepted in social science research. However, the findings may not be as representative as findings based on probability samples. As such, caution should be adopted in interpreting the results.

Notwithstanding the limitations, we would like to highlight that social media has become a primary news source for millions worldwide. The COVID-19 pandemic has witnessed a flood of conspiracy theories regarding vaccines and their efficacy. Within this setting, the current study recommends to public policymakers and vaccine communication strategists that any attempt to reduce vaccine hesitancy in society should factor in the adverse effects of social media news use that can increase vaccine safety concerns. Social media companies should enforce more robust content moderation on their platform to safeguard the public against mis- and disinformation regarding COVID-19 vaccines. Attempts should not be limited to curtailing COVID-19 vaccine-related mis- and disinformation on social media platforms, but an equal effort should be paid to educate the public regarding news literacy. That said, future scholars should examine the discussed framework within other contexts, especially in settings with lower levels of news literacy, to examine if the effects of social media news use on vaccine hesitancy (through skepticism) are more widely applied.

## Data Availability Statement

The raw data supporting the conclusions of this article will be made available by the authors, without undue reservation.

## Ethics Statement

The studies involving human participants were reviewed and approved by the Institutional Review Board at Nanyang Technological University. The patients/participants provided their written informed consent to participate in this study.

## Author Contributions

SA designed the study, analyzed the data, and wrote the manuscript. MR and JC analyzed the data and wrote the manuscript. All authors approved the submitted version.

## Conflict of Interest

The authors declare that the research was conducted in the absence of any commercial or financial relationships that could be construed as a potential conflict of interest.

## Publisher’s Note

All claims expressed in this article are solely those of the authors and do not necessarily represent those of their affiliated organizations, or those of the publisher, the editors and the reviewers. Any product that may be evaluated in this article, or claim that may be made by its manufacturer, is not guaranteed or endorsed by the publisher.
