# Supplementary material for: Social Media News Use Induces COVID-19 Vaccine Hesitancy Through Skepticism Regarding Its Efficacy: A Longitudinal Study From the United States
Source: Front Psychol. 2022 Jun 10;13:900386. doi: 10.3389/fpsyg.2022.900386 (PMC9226607; doi:10.3389/fpsyg.2022.900386)
Supplement: Supplementary file 1 [file Table_1.DOCX]

**Supplementary File**

**Details of Controls**

News trust was measured by three items asking respondents how much (1 = not at all to 5 = extremely) do they trust the news and information about politics and public affairs that they get from a) national news organizations b) local news organizations, and c) international news organizations (*mean* = 2.69, *SD* = 1.14, α = .90).

Political interest was measured by a single question asking the respondents how interested (1 = not at all to 5 = extremely) individuals are in politics (*mean* = 3.21, *SD* = 1.23).

Political trust was measured by asking respondents how much (1 = not at all to 7 = a lot) they trust the government (*mean* = 3.89, *SD* = 1.79).

Political efficacy was measured via two items asking respondents to what extent do you agree or disagree (1 = strongly disagree to 7 = strongly agree) with the following statements a) I have a good understanding of the important political issues facing our country, and b) I think I am better informed about politics and government than most people (*mean* = 4.96, *SD* = 1.37, Spearman-Brown coefficient = .77).

Partisanship was measured on a seven-point scale ranging from 1 = strong democrat to 7 = strong republican (*mean* = 4.01, *SD* = 2.11).

Traditional media news use was created by asking the respondents how frequently (1 = never to 5 = daily) they consumed news about politics and public affairs from three mediums; a) television, b) print newspapers, and c) radio. The responses to these three items were averaged to create a scale of traditional media news use (*mean* = 2.89, *SD* = 1.14, α = .65).

**Moderated Mediation Results**

Moderated mediation results for social media news use on vaccine hesitancy through skepticism regarding vaccine efficacy with news literacy as moderator. ^a-b^

| Mediator | News Literacy | Indirect Effects | Std Error | 95% Confidence Interval |
| --- | --- | --- | --- | --- |
| Skepticism regarding vaccine efficacy | -1 Std Dev | .102 | .023 | .058 to .148 |
|  | Mean | .070 | .018 | .06 to .105 |
|  | +1 Std Dev | .038 | .024 | -.012 to .084 |

^a-b.^ Analyses were performed using the PROCESS macro for SPSS (Model 8; Hayes 2018), applying 5,000 bootstrapped bias-corrected resample. Statistical controls include age, gender, education, income, race, political trust, political interest, political efficacy, partisanship, traditional media news us, news trust, and news literacy.
